# Single-Molecule Trapping and Measurement in a Nanostructured Lipid Bilayer System

**DOI:** 10.1021/acs.langmuir.2c02203

**Published:** 2022-11-03

**Authors:** Maria Bespalova, Robin Öz, Fredrik Westerlund, Madhavi Krishnan

**Affiliations:** † Physical and Theoretical Chemistry Laboratory, Department of Chemistry, University of Oxford, South Parks Road, OxfordOX1 3QZ, United Kingdom; ‡ Department of Biology and Biological Engineering, Chalmers University of Technology, 412 96Gothenburg, Sweden; § The Kavli Institute for Nanoscience Discovery, Sherrington Road, OxfordOX1 3QU, United Kingdom

## Abstract

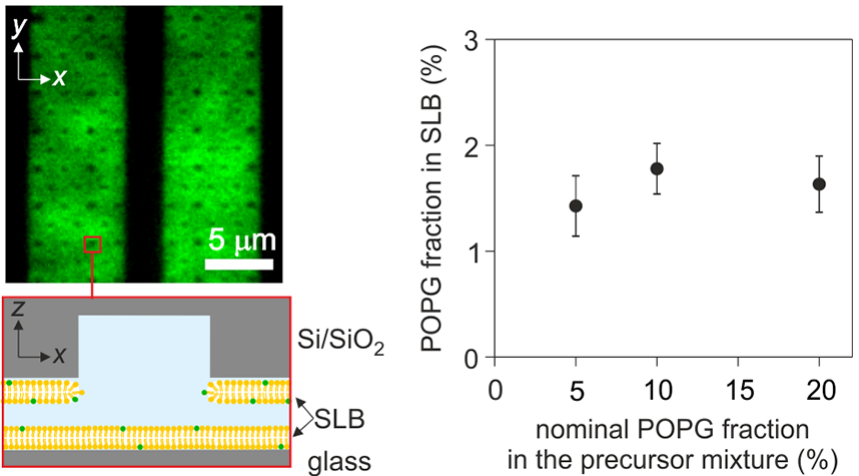

The repulsive electrostatic force between a biomolecule and a like-charged surface can be geometrically tailored to create spatial traps for charged molecules in solution. Using a parallel-plate system composed of silicon dioxide surfaces, we recently demonstrated single-molecule trapping and high precision molecular charge measurements in a nanostructured free energy landscape. Here we show that surfaces coated with charged lipid bilayers provide a system with tunable surface properties for molecular electrometry experiments. Working with molecular species whose effective charge and geometry are well-defined, we demonstrate the ability to quantitatively probe the electrical charge density of a supported lipid bilayer. Our findings indicate that the fraction of charged lipids in nanoslit lipid bilayers can be significantly different from that in the precursor lipid mixtures used to generate them. We also explore the temporal stability of bilayer properties in nanofluidic systems. Beyond their relevance in molecular measurement, such experimental systems offer the opportunity to examine lipid bilayer formation and wetting dynamics on nanostructured surfaces.

## Introduction

Supported lipid bilayers (SLBs) are self-assembled two-dimensional thin-film coatings composed of a single phospholipid bilayer resting on or near a solid support.^1^ The ability of SLB systems to mimic native cell membranes has been successfully employed in fundamental biophysical studies for several decades.^2,3^ Specifically, SLB systems serve as a useful tool for studying cell adhesion and repulsion, peptide–cell interactions, cell–surface interactions, and membrane properties in general.^4−12^ Given their unique properties, such as biological inertness, amphiphilicity, and ability to serve as matrixes for surface immobilization, SLBs have found applications in biosensors, drug delivery, and medical diagnostics.^13−15^ Furthermore, ongoing advances in microfluidics, biosensor design, and micro- and nanofabrication have opened up new application avenues for nanostructured SLBs.^16−18^ Owing to their antifouling properties and the diversity in lipid species chemistry, SLBs have been used to modulate surface properties at the nanoscopic level.^19−24^ In this work, we explore the relevance of lipid bilayer coatings for electrostatic trapping of single molecules in solution. Such coatings are advantageous in that they offer tunability of both the sign and magnitude of the surface electrical charge in device-based systems where surface interactions play a strong role.^25^

**Figure 1:**
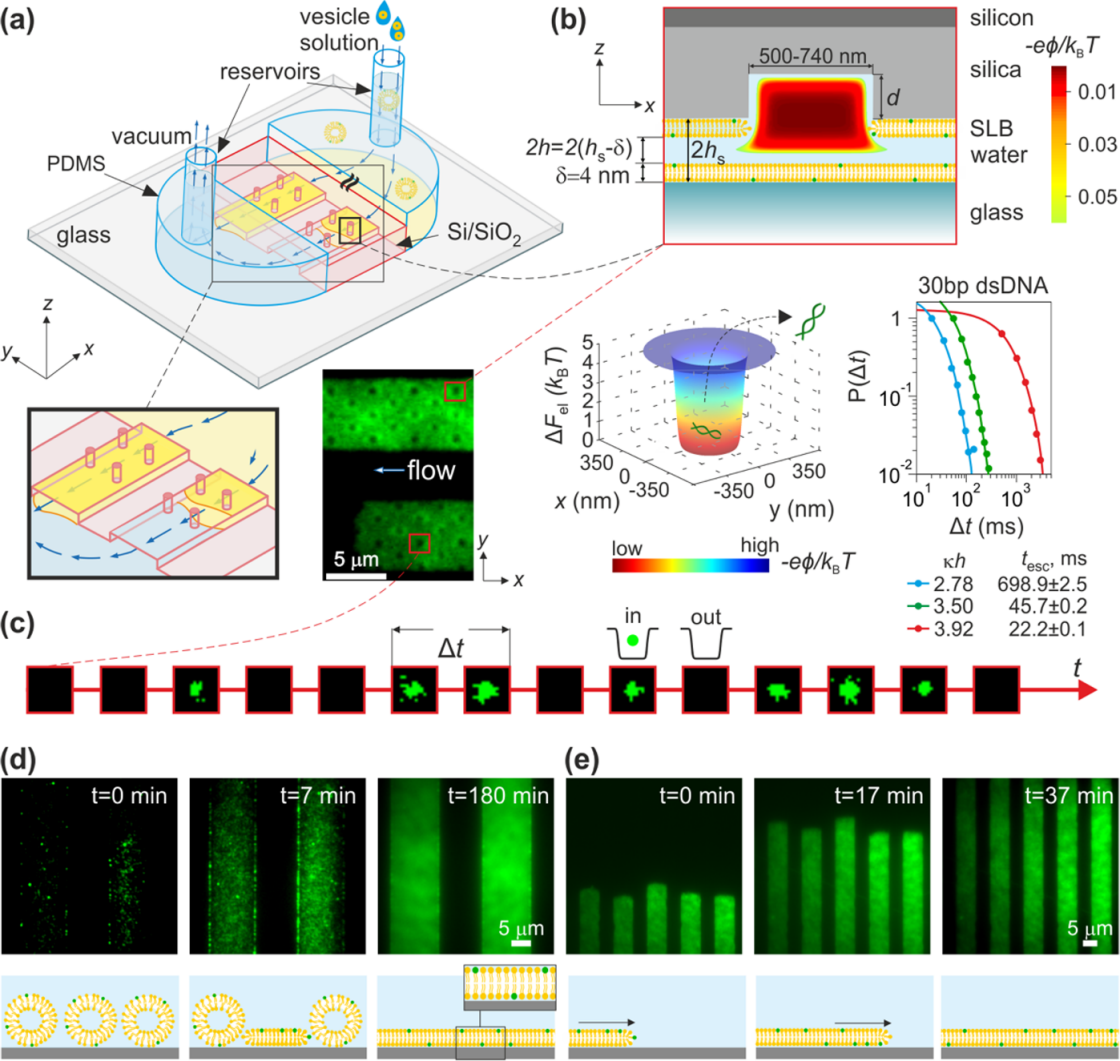
(a) Schematic illustration of a single-molecule trapping device consisting of a parallel array of nanoslits whose surfaces are passivated with SLBs (yellow). Bottom left: illustration of SLBs spreading in the nanoslits carrying surface indentations, or “pockets”, on the top surface. The dimensions are not to scale. Bottom right: wide-field fluorescence image of SLBs spreading in the nanostructured region of two nanoslits (top view). (b) Cross section of a single trapping nanostructure or pocket depicting the spatial distribution of electrical potential in the nanostructured region. Nanostructured features were ≈160 nm deep and either ≈500 or 740 nm in diameter. The SLBs coating the nanoslit surfaces contain POPG/POPC (yellow) and a small fraction of fluorescent Rhodamine-DHPE (green). Bottom left: calculated spatial distribution of minimum axial electrostatic free energy, Δ*F*_el_. Bottom right: Probability density distribution, *P*(Δ*t*), of measured escape times, Δ*t*, plotted on log–log scale for measurements on 30bp dsDNA in nanostructured POPG/POPC SLB system at three different values of dimensionless slit depth, *κh*, and fitted to the expression *P*(Δ*t*) ∝ exp (−Δ*t*/*t*_esc_)/*t*_esc_. (c) A series of snapshots in a trap displays the duration of a single recorded escape time, Δ*t*, for fluorescently labeled single molecules entering and leaving a single trap location. Note that although we use fluorescently labeled lipids to visualize SLB formation as shown in (a), (d) and (e), the SLB is bleached to eliminate the fluorescent background signal in single-molecule measurements. This gives rise to a bright signal from the molecule visible against a dark device background. (d) Top: wide-field fluorescence images of SLB formation in ≈10 μm wide slits recorded over 3 h while flushing the SUV suspension containing nominally 20% of POPG at pH 9 and *c* = 100 mM. Bottom: schematic representation of SLB formation by vesicle rupture and fusion at the surface. (e) Top: wide-field fluorescence images of SLBs in ≈5 μm wide slits created by “steady-migration”, recorded within 37 min while flushing the SUV suspension nominally containing 20% of POPG at pH 3.5 and *c* = 100 mM. Bottom: schematic representation of SLB migrating steadily in the direction of the flow indicated by the arrow.

Our experimental approach exploits the electrostatic fluidic trap described in detail in previous work.^26−29^ The electrostatic fluidic trap relies on the equilibrium thermodynamic repulsion experienced by an electrically charged object confined in a fluid-filled gap between two like-charged parallel plates that form a slit. Geometric tailoring of one of the parallel plates with nanoscale surface indentations, or “pockets”, leads to a local interaction energy minimum, creating a stable thermodynamic spatial trap for a charged object in solution (Figure 1a,b).

In our previous work, the walls of the nanoslits were composed of glass and silicon dioxide. Both materials acquire a negative charge in aqueous solutions due to dissociation of terminal silanol groups: SiOH ⇌ SiO^–^ + H^+^ (ref (30)). Although silicon dioxide is an established material for microfluidic and nanofluidic device fabrication, its surface properties can pose restrictions on experimental conditions used for electrostatic trapping.^31^ Surface silanol groups are known to exist in states with different values of acid dissociation constants, p*K*_a_, ranging from 2.3 to 10.7 with an average of ≈7 (refs (32, 33)). Moreover silicon dioxide surface chemistry even displays strong dependence on treatment history.^32^ Due to the relatively high value of the average p*K*_a_ we performed all previous single-molecule trapping experiments at pH 9 to ensure that the surface carried substantial charge density (≈−0.1 *e* /nm^2^) (ref (34)). Doing so permitted stable electrostatic trapping ranging from tens of milliseconds for single organic fluorophore molecules carrying a single elementary charge to tens of minutes for highly charged proteins such as Stml-1 (*q*_eff_ ≈ −89 *e*).^35,38^ However, high pH may not prove optimal for measurements on some biomolecular species, e.g., single-stranded RNA, which tends to degrade in alkaline solutions.^36^

In this study, we demonstrate electrostatic trapping and effective charge measurements on single molecules in nanostructured slit systems whose surfaces are passivated using SLBs carrying net electrical charge. Electrostatic free energies associated with the molecule–surface interaction are inferred from experimentally measured potential-well depths in the molecular trapping process as described in detail in previous work and recapitulated briefly in the following section.^35,37−40^ In earlier studies, we worked with silicon dioxide surfaces and used measured free energies inferred for charged molecular species in order to infer their effective charge, *q*_eff_ (refs (35, 37−41)). In this study, we use double stranded DNA molecules of known effective charge in order to infer the surface electrical properties of supported lipid bilayers. We report measurements of the surface electrical potential and net charge density as a function of lipid bilayer composition. Since the phosphodiester group in phospholipids is highly acidic in nature (p*K*_a_ ≤ 3.5), the use of SLBs should support trapping of negatively charged molecules in aqueous solutions where pH ≥ 4. Using the escape-time electrometry (ET*e*) approach, we also explore the possibility of tuning surface charge by varying the fraction of charged lipids in SLBs and measuring the depth of the potential well of the corresponding electrostatic trap.

### Brief Description of the Electrostatic Fluidic Trap and the ET*e* Measurement Principle

We optically visualize and measure the strength of electrostatic repulsions between a charged molecule and like-charged probe surfaces in solution using the recently developed ET*e* approach, which relies on wide-field optical microscopy.^35,37−41^ ET*e* measures the reduction in system free energy associated with transferring a charged molecule from a gap between like-charged parallel plates into a local nanostructured indentation region in the gap. This “trap” region is zone of weak axial confinement where the molecule-plate repulsion is negligible compared to the parallel-plate region^26^ (Figure 1a,b). Typically, an array of such electrostatic fluidic traps is created using periodic nanostructured indentations in one surface of a parallel plate slit composed of silica surfaces separated by a gap of height 2*h* ≈ 75 nm. We introduce an aqueous suspension of the molecular species of interest, labeled with exactly 2 fluorescent ATTO 532 dye molecules, at a concentration of 50–100 pM into a system with multiple parallel lattices of traps (Figure 1a). In this work, we report on measurements performed on double stranded DNA molecules in nanoslits whose silicon oxide/glass walls are passivated with supported lipid bilayers of various lipid compositions.

The ET*e* technique measures the average time, *t*_esc_, taken by a molecular species to escape from an electrostatic fluidic trap of typical depth, *W* (Figure 1b,c). Imaging the escape dynamics of trapped single molecules in a lattice of traps enables us to extract individual molecular escape events of duration, Δ*t*, in each trap (Figure 1c). Fitting the probability density distribution, *P*(Δ*t*), of measured escape times, Δ*t*, to the expression of the form *P*(Δ*t*) ∝ exp (−Δ*t*/*t*_esc_)/*t*_esc_ permits us to extract the value of the average escape time, *t*_esc_. In this work, we typically have *N* ∼ 10^3^–10^4^ escape events. Since escape times are exponentially distributed, the fractional measurement uncertainty on *t*_esc_, given by , is about 1–3% (ref (35)). For *W* > 4*k*_B_*T*, the relationship between *t*_esc_ and *W* is well described by Kramer’s relation:
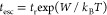
1where *t*_r_ represents a position relaxation time of the molecule.^42^ In order to convert measured *t*_esc_ values to the depth of the underlying potential well, *W*, we perform Brownian Dynamics simulations of the escape process in a lattice of traps.^37^ As we have shown in our previous work, *W* = Δ*F*_el_ + *f* is composed of two parts: the electrostatic interaction free energy, Δ*F*_el_, and an entropic contribution to positional fluctuations of the molecule, *f* (ref (38)). We determine the entropic contribution as previously described and extract Δ*F*_el_ for each measurement. In turn, we have also shown that Δ*F*_el_ = *q*_eff_ ϕ_m_, where *q*_eff_ is the effective charge of the molecule in solution and ϕ_m_ denotes the electrical potential at the midplane of the slit.^43^ Further, ϕ_m_ can be well approximated using the linear superposition formula:
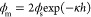
2where ϕ_s_ is an effective surface potential, and *κh* is a dimensionless slit depth, where  denotes the Debye length for a monovalent salt solution of a concentration, *c* (in M), and *h* = *h*_s_ – δ. Here 2*h*_s_ is the slit height measured by AFM and δ = 4 nm is the thickness of a single lipid bilayer (Figure 1b) determined by small angle neutron and X-ray scattering data and inferred from molecular dynamics simulations.^44−49^ Since the true height of the slit is not known to within a few nanometers, the results will not be affected from the same level of uncertainty on the lipid bilayer thickness. Equation 2 is based on a superposition approximation using the linearized Poisson–Boltzmann (PB) equation. Equation 2 is in excellent agreement with the results of the full nonlinear PB equation (NLPB) far away from the slit walls and has been used extensively in previous work.^35,37−41^ Disparities between the electrical potential profile calculated using the NLPB equation and that given by the linear superposition approximation are only apparent in the region within a few nanometers of the slit surfaces. Because this region is rarely sampled by a like-charged molecule, the linear superposition approximation shown in eq 2 is sufficiently accurate. Note that ϕ_s_ can equal the “true surface potential”, say ϕ_s,0_, in the regime of very small values, |*eϕ*_s,0_| ≪ 25 mV.

As reflected in eq 1, the measurand *t*_esc_ depends exponentially on the effective charge of the molecule, *q*_eff_. As shown in our previous work, the fractional measurement uncertainty on *q*_eff_ is given by *t*_esc,e_/Δ*F*_el_ (ref (35).). Thus, 1–3% uncertainty on *t*_esc_ would result in ≈0.3–1% measurement uncertainty in *q*_eff_. In this study, we work with dsDNA and fluorescent dye molecules whose effective electrical charge values are known both from calculations and extensive investigations in silicon dioxide-based systems in previous work.^35,37,38,40,43^ Therefore, the only unknown quantity in these experiments is the effective surface potential, ϕ_s_, of the SLBs coating the slit surfaces.

## Experimental Section

### Fabrication of Nanofluidic Trapping Devices

The devices were fabricated using Silicon/Silicon dioxide and glass substrates as previously described.^26,35,37−39^ Briefly, the device fabrication procedure consisted of deep-UV lithography to pattern the nanoslit regions and electron beam lithography in order to define nanostructured trap regions or “pockets”. In each case, lithography was followed by reactive-ion etching to etch both the slit and pocket features into the silicon dioxide substrate surface. The dimensions of the channels and pockets were measured using scanning electron microscopy (SEM), atomic force microscopy (AFM), and profilometry. We used nanoslits of height 2*h*_s_ = 71–78 nm and width of ≈5 and ≈10 μm, and pockets of depths *d* ≈ 160 and ≈290 nm and radii, *r* ≈ 250, ≈ 300, and ≈370 nm. (Figure 1b).

### Vesicle Preparation

Vesicles were prepared by the thin-film hydration method followed by extrusion. We used 1-palmitoyl-2-oleoyl-glycero-3-phosphocholine (POPC) and sodium salt of 1-palmitoyl-2-oleoyl-*sn*-glycero-3-phospho-(1′-rac-glycerol) (POPG) dissolved in chloroform (Avanti Polar Lipids). Fluorescently labeled lipid N-(Lissamine rhodamine B sulfonyl)-1,2-dihexadecanoyl-*sn*-glycero-3-phosphoethanolamine, triethylammonium salt (Rhodamine-DHPE) (Biotium, Inc.) was dissolved in chloroform to a final concentration of 5 mg/mL.

In order to optimize the lipid bilayer coating procedure in our nanostructured fluidic slit system, we prepared the precursor lipid mixtures by mixing POPC, POPG, and Rhodamine-DHPE lipids in a ratio of 79:20:1 mol % respectively (Figure 1d,e). For ET*e* measurements, lipids in precursor lipid mixtures were mixed in the following ratios: 95:5, 90:10, and 80:20% of POPC:POPG with 10–6% Rhodamine-DHPE. Note that we used a very low concentration of labeled lipids in bilayers prepared for ET*e* measurements. This permits bilayer formation to be observed and confirmed while ensuring that bilayer fluorescence can be completely bleached prior to single-molecule measurements. Precursor lipid mixtures contained 10 mg of lipids in total.

Once the precursor lipid mixtures were prepared, chloroform was evaporated with a stream of nitrogen followed by vacuum desiccation for at least 4 h. Multilamellar vesicles (MLVs) were prepared by lipid thin-film rehydration in lipid buffer (10 mM Tris-HCl pH 8, 10 mM Boric acid, 0.225 mM EDTA, 100 mM NaCl) to a final lipid concentration of 1 mg/mL and vortexing for at least 5 min afterward. The MLVs were further extruded using Avanti Mini Extruder (Avanti Polar Lipids). To prepare small unilamellar vesicles (SUVs), we subsequently extruded MLVs 21 times through a 50 nm pore-size pore membrane and 31 times through a 30 nm membrane. We further diluted freshly extruded SUV suspensions with lipid buffer to a final concentration of 250 μg/mL, titrated with HCl to the desired pH value, and we used the suspension immediately for coating of nanoslits.

### Supported Lipid Bilayer (SLB) Formation

In order to form the SLB on nanoslit surfaces, we used a microfluidic setup illustrated in Figure 1a. Prior to loading the SUV suspension, the slits were flushed with lipid buffer (10 mM Tris-HCl pH 8, 10 mM Boric acid, 0.225 mM EDTA, 100 mM NaCl) for 15–20 min using pressure driven flow. The extruded vesicle suspension was then flushed through the slits for at least 12 h. Visualization of SLB formation was achieved by fluorescence excitation of Rhodamine-DHPE. Once the lipid bilayer coated the slits entirely, the flow was arrested and plain lipid buffer was flushed into the nanoslits in both flow directions to remove any debris and intact vesicles from the slits. Upon completion of the SLB formation, the fluidity and continuity of the lipid bilayer was confirmed using fluorescence recovery after photobleaching (FRAP).

### Purification of DNA Samples

All DNA fragments were purchased from IBA Lifesciences (Germany) with a single ATTO 532 dye molecule coupled to both 5′ termini. The oligomers were purified with RP-HPLC using a Reprosil-Pur 200 C18 AQ column (Dr. Maisch, Germany) and elution with a gradient of acetonitrile in an aqueous 0.1 M triethylammonium acetate solution.

### Imaging

Optical measurements were performed using widefield fluorescence imaging. Fluorescence excitation was achieved by illuminating Rhodamine-DHPE (for observing the SLBs) or ATTO 532-labeled DNA and ATTO 542 dye molecules (in escape time electrometry measurements) with a 532 nm DPSS laser (MGL_III-532_100 mW, PhotonTec, Berlin) that was focused at the back aperture of a 60×, NA = 1.35 oil immersion objective (Olympus, U.K.). Images were acquired using a sCMOS camera (Prime95B, Photometrics).

### ET*e* Measurements

Prior to ET*e* measurements, the slits were flushed with 1 mM NaCl aqueous solution for at least 30 min. Then the flow was arrested, and the system was allowed to equilibrate for 20–30 min before loading the molecular species of interest. Prior to molecular loading for escape-time measurements, the fluorescent signal due to the Rhodamine-DHPE in the bilayers coating the slit surfaces was bleached in the entire measurement region. Images of molecular motion in the trap arrays were then acquired over a period of about 10–20 min per sample.

## Results and Discussion

**Figure 2:**
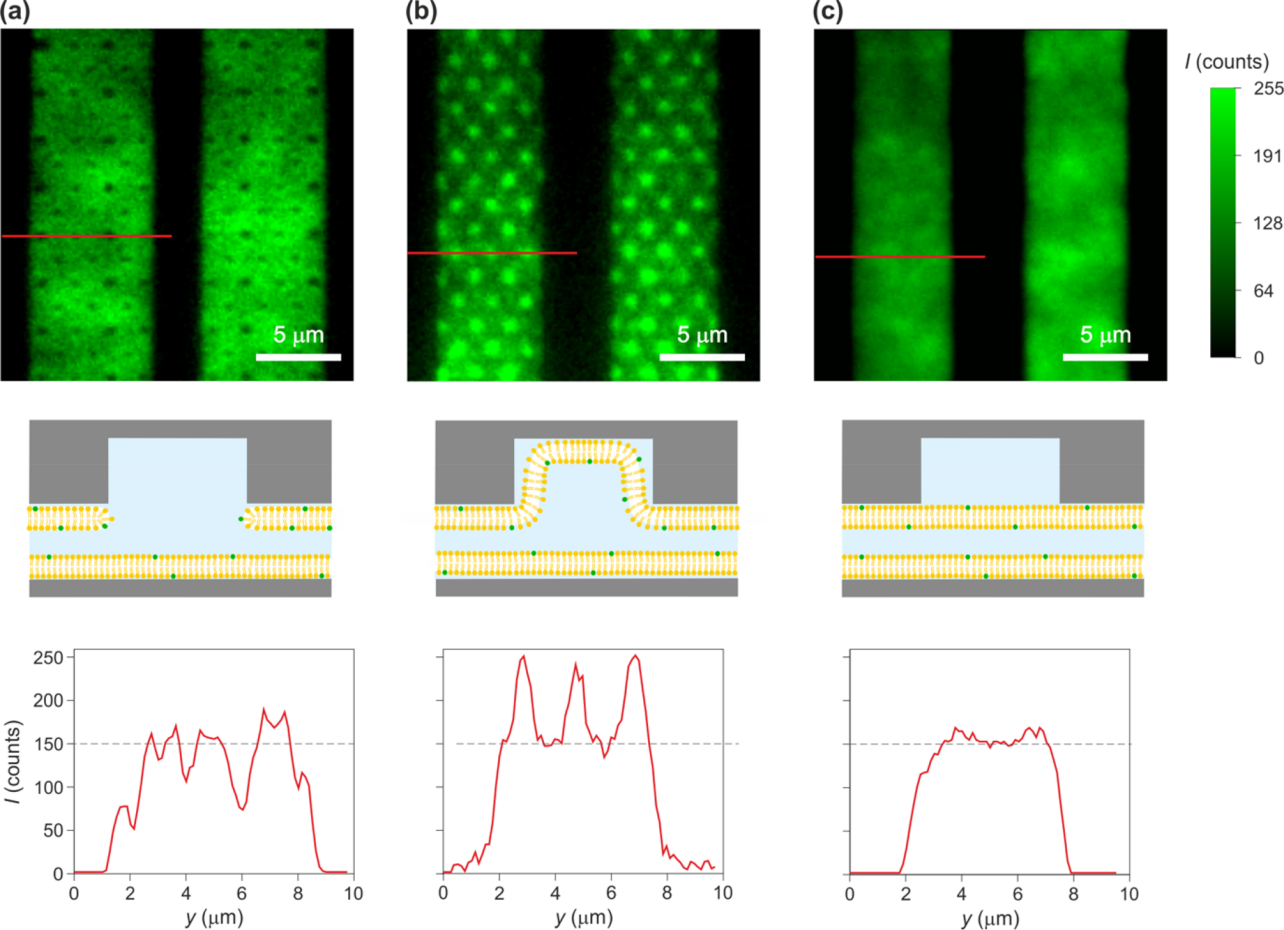
Various modes of surface coverage of SLBs in nanostructured silicon dioxide slits. Top panels depict the wide-field fluorescence images of passivated slits upon the completion of SLB formation when using SUV suspensions with (a) pH 3.5, *c* = 100 mM (See Supporting Movie), (b) pH = 3.5, *c* = 2 M, and (c) pH = 9, *c* = 100 mM. Central panels illustrate possible SLB coverage scenarios in a single pocket region for each of the illustrated fluorescence images: the SLB (a) circumvents the pocket, (b) coats the pocket, and (c) caps the pocket (illustration not to scale). Bottom panels depict intensity profiles plotted along the red lines drawn on top of the corresponding wide-field fluorescence images. Gray dashed line indicates *I* = 150 counts and shows that intensity level at the planar regions of the slits is relatively constant across the three scenarios.

**Figure 3:**
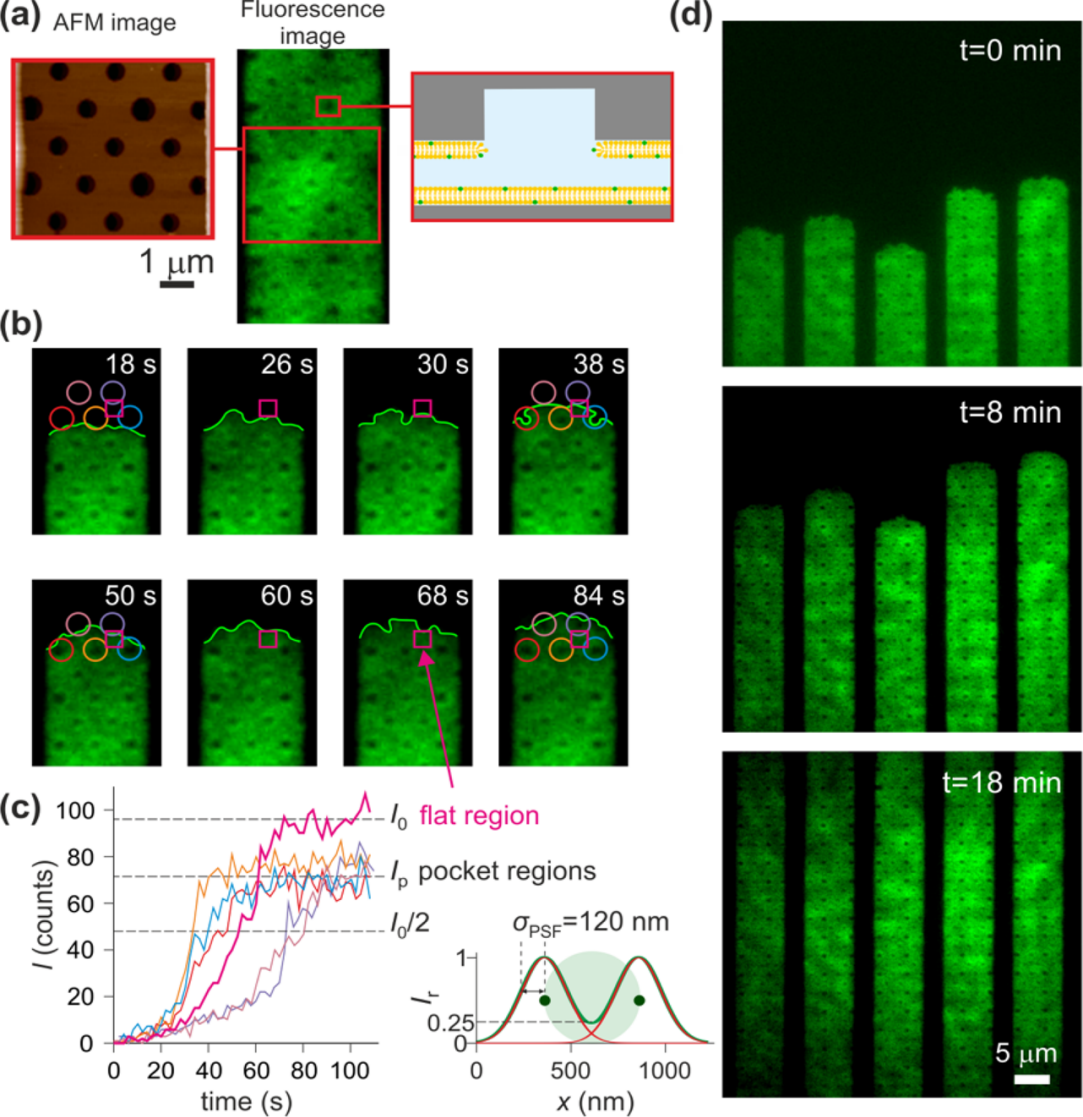
Steadily migrating SLBs circumvent nanostructured surface features. (a) AFM image of the nanostructured slit surface with circular “pocket” indentations (left). Wide-field fluorescence image of the pocket region upon the completion of SLB formation (middle). Here, we used the device with ≈160 nm deep pockets of two different radii, *r* ≈ 250 and ≈370 nm. Schematic representation of a cross section through the center of a single pocket where the lipid bilayer coats the slits but circumvents the surface feature (right). (b) Wide-field fluorescence images of progressing SLB sequentially recorded in a ≈ 5 μm wide slit while flushing the SUV suspension nominally containing 20% of POPG with pH 3.5 and *c* = 100 mM. Colored circles indicate the pocket regions traversed by the lipid bilayer during the recording, and the square displays a flat region of the slit in between two neighboring pockets (See Supporting Movie). (c) Temporal evolution of fluorescence intensity signal, *I*, in the pockets and flat regions during traversal of the SLB across the slit as shown in (b) (left). *I*_0_ and *I*_p_ denote the average intensities in the flat region and pocket regions upon the completion of the SLB formation, respectively. Sketch of optical point spread functions (PSFs) for two fluorescent Rhodamine-DHPE molecules located on the circumference of the surface nanostructure in an SLB coated slit (green circle) (right). For σ_PSF_= 120 nm and a pocket radius of 250 nm the fluorescence signal intensity in the center of the pocket is ≈25% of the peak intensity at the location of each molecule due to lateral overlap of the PSFs from the two diametrically opposite molecules on the pocket circumference. (d) Wide-field fluorescence images sequentially recorded within 18 min of the SLBs in ≈5 μm wide slits while flushing the SUV suspension nominally containing 20% of POPG with pH 3.5 and *c* = 100 mM at a typical speed of ≈80 μm/s.

In order to construct a nanostructured SLB system, we used a microfluidic setup illustrated in Figure 1a. We first created a suspension of small unilamellar vesicles (SUVs) containing zwitter-ionic lipid 1-palmitoyl-2-oleoyl-glycero-3-phosphocholine (POPC), negatively charged lipid sodium salt of 1-palmitoyl-2-oleoyl-*sn*-glycero-3-phospho-(1′-rac-glycerol) (POPG), and a small amount (≤1 mol %) of Rhodamine-DHPE by extrusion of a multilamellar lipid vesicle suspension through polycarbonate membranes with a 30 nm pore diameter (Experimental Section). Inclusion of fluorescently labeled lipid Rhodamine-DHPE in the lipid mixture enabled visualization of the SLB formation process (Figures 1d,e, 2, and 3). We loaded POPG/POPC SUVs in the inlet reservoir of the system and flushed the suspension through nanoslits of height 2*h*_s_ ≈ 75 nm using pressure driven flow at a typical speed of 80 μm/s. We found that loading larger vesicles, prepared by extrusion through polycarbonate membranes with larger pore diameters (≥50 nm), led to clogging of the nanoslits.

### Mechanisms of Spreading of Supported Lipid Bilayers on Nanostructured Surfaces in a Fluidic Slit

We observed two different mechanisms of SLB formation in our nanoslits depending on the pH and ionic strength of the SUV suspension loaded into the slits (Figure 1d,e). Figure 1d presents a scenario in which vesicles entered the slits, settled down on the surface, and ruptured within an hour, forming discrete patches of lipid bilayer. Subsequently, these bilayer patches coalesced into a continuous lipid bilayer, ultimately coating the slits entirely. We observed this mechanism of lipid bilayer formation for SUV suspensions with pH ≥ 8 and NaCl concentration, *c* = 100 mM, in ≈5 and ≈10 μm wide nanoslits. In contrast, Figure 1e, depicts a different, steady-migration mechanism of SLBs formation in the direction of fluid flow through the nanoslits. This mechanism was observed when using SUV suspensions of pH 3.5 and *c* = 100 mM–2 M in ≈5 μm wide nanoslits. We found that the latter set of conditions, i.e., pH 3.5 and *c* = 100 mM–2 M, provided more reproducible SLB behavior in our experiments. When using SUV suspensions with higher pH values in ≈5 μm wide slits, we did occasionally observe steady migration behavior with a progressing SLB front as in Figure 1e. However, under no experimental conditions did we observe the steady-migration mechanism of SLB formation in ≈10 μm wide nanoslits. For all measurements presented in this work, we therefore used ≈5 μm wide nanoslits and the steady-migration SLB formation procedure depicted in Figure 1e.

We further examined the steady-migration mechanism of SLB formation in regions of the fluidic slit carrying nanostructured surface indentations (Figures 2 and 3). It has been previously demonstrated that the spreading behavior of lipid bilayers over a surface with nanostructured indentations depends on the size of the vesicles as well as on the pH and salt concentration of the bulk solution.^20,50,51^ Inspired by these observations, we probed the influence of both pH and ionic strength of a SUV suspension on the spreading behavior of negatively charged SLBs in our nanostructured silicon dioxide slits. For these experiments, we used devices with nanostructure indentations of depth ≈160 nm and with two different radii, *r* ≈ 250 and ≈370 nm. We probed different SUV suspensions in a range of pH from 3.5 to 9 and NaCl concentrations, *c*, in a range from 100 mM to 2 M. Figure 2 illustrates three scenarios observed once the lipid bilayer front had traversed a slit surface with an array of nanostructured indentations. When using POPG/POPC SUV suspensions (containing nominally 5–20 mol % of POPG) with *c* = 100 mM and pH 3.5, we observed a ≈25% decrease of fluorescence signal at the pocket locations, *I*_p_, compared to the intensity, *I*_0_, at the feature-free flat surface region, i.e., *I*_p_/*I*_0_ ≈ 0.75 (Figures 2a (top) and 3). Note that the fluorescence signal in the feature-free region of the slit in our experiment contains contributions from both the top and bottom surfaces and that nanostructured pocket features that disrupt the continuity of the bilayer are only present on one surface of the slit. A naive estimate of the intensity in an uncoated pocket region would therefore suggest *I*_p_/*I*_0_ ≈0.5. However, since the nanostructure radii, *r*, in our devices are in the range of 250–370 nm, which is comparable to the width of the optical point spread function (PSF) (σPSF= 120 nm in our system), we expect the fluorescence signal in the center of the pocket regions to also contain contributions from the fluorescent lipid bilayer at the circumference of the structure (as illustrated in Figure 3c). Thus, the ratio *I*_p_/*I*_0_ ≈ 0.75 < 1 likely points to a scenario where the lipid bilayer circumvents the pockets as illustrated in Figures 2a (central) and 3a (see Supporting Movie).

Further, using SUV suspensions containing higher NaCl concentrations in the range *c* = 200 mM-2 M and pH 3.5, we occasionally observed a ≈70% higher fluorescence intensity at the pocket locations in comparison to the featureless region, i.e., *I*_p_/*I*_0_ ≈ 1.7 (Figure 2b, top). As discussed in ref (20), a ratio of *I*_p_/*I*_0_ ≈ 1.7 > 1 likely reflects a scenario where the lipid bilayer follows the contour of the pockets as illustrated in Figure 2b (central). The enhanced intensity at the pocket locations may be simply explained by the fact that pocket diameter (500 and 740 nm) is on the order of the wavelength of light. When the vertically oriented pocket walls are conformally coated with a fluorescent bilayer, the number of fluorescent emitters per unit area projected on to the 2D image plane is much higher than in the planar regions. This gives rise to bright looking pocket regions. Note that in an idealized situation of infinite spatial resolution in imaging, the pockets would appear to have bright rings surrounding central lower intensity disc regions with similar intensity to the flat slit regions. We further found that it was challenging to obtain controllable and reproducible results under these conditions with the SLB occasionally circumventing the pocket nanostructures, as observed in solutions of *c* = 100 mM NaCl described previously.

Finally, Figure 2c depicts a scenario observed for SLBs formed in a solution with pH 9 and *c* = 100 mM. Here we found no optical evidence of the presence of nanoscale surface topography upon completion of SLB spreading, with the fluorescence intensities in the pocket and flat regions displaying no obvious spatial disparities. This suggests that under these conditions the SLB simply traverses a nanostructured pocket, neither circumventing it nor coating its surface, but rather capping the indentation as shown in Figure 2c (central). Previous studies have reported this regime of coating behavior under experimental conditions similar to our study, i.e., pH 9 and *c* = 100 mM (ref (20)). Note that the level of background intensity at the planar regions of the slits remains roughly constant across the three scenarios, notwithstanding some amount of spatial nonuniformity in the fluorescence signal from the SLBs, the exact origin of which remains unclear (Figure 2, bottom).

Overall, we observed slightly different SLB spreading behavior than that described in previous work involving feature diameters 3–4 times smaller than in this study.^20^ In previous studies, lipid bilayers were found to conformally coat the pocket surfaces at pH 8, whereas our experiments reveal such behavior only occasionally in solutions with pH 3.5 and in the presence of up to 2 M NaCl. It is evident that a low solution pH and strong electrostatic screening favor the formation of conformal SLB coatings on like-charged nanostructured surfaces. It is also clear that increased electrostatic repulsion between the bilayer and silicon dioxide surface at higher pH can introduce interesting modes of wetting behavior when a moving bilayer encounters a surface defect presented by a nanostructured indentation. Since electrostatic interactions play a strong role in the spreading behavior of a charged SLB on a silicon dioxide surface, the process is not only strongly affected by pH and ionic strength of the SUV suspension but can also be highly dependent on sample-surface properties.

For all trapping experiments, described further in this work, we passivated our nanoslit surfaces using the approach depicted in Figures 2a and 3 (see Supporting Movie). Since ET*e* measurements rely on recording the emitted fluorescence signal from trapped fluorophore-labeled molecules, we reduced the fraction of labeled lipids, Rhodamine-DHPE, in the precursor lipid mixtures down to 10^–6^%. A low concentration of labeled lipids supported the observation of the moving front of the SLB and enabled us to verify the continuity of the SLB upon completion of bilayer formation. Prior to every ET*e* measurement, we bleached the fluorescence signal from Rhodamine-DHPE in order to eliminate any bilayer fluorescence in the observation of trapped single molecules in solution. All trapping experiments described below were performed in nonbuffered aqueous solutions (pH 5–6) containing ≈1 mM NaCl.

### Using ET*e* to Probe the Effect of Charge Content in SLBs on Molecule–Surface Electrostatic Interactions

**Figure 4:**
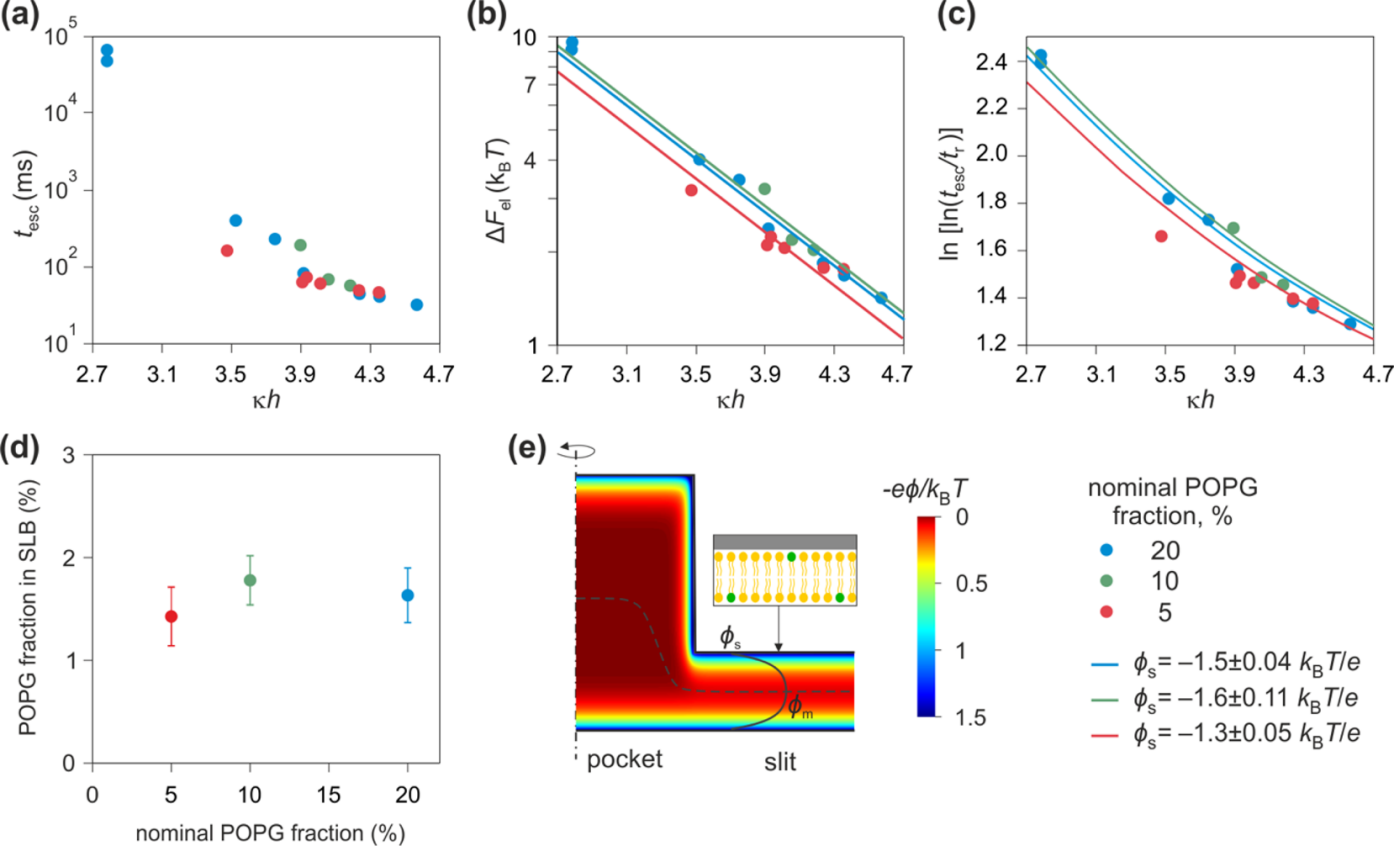
Trapping 60bp dsDNA (*q*_eff_ = −44.8 *e*) molecules in SLB systems nominally containing 5% (red), 10% (green), and 20% (blue) of POPG. The three experiments with nominally different SLBs were performed sequentially in the same trapping device. (a) Dependence of measured escape times, *t*_esc_, on dimensionless slit height, *κh*. (b) Measured values (symbols) for electrostatic free energy Δ*F*_el_ fitted to eq 3 with a single fit parameter, ϕ_s_, the effective surface electrical potential of the SLB. We obtained ϕ_s_ = −1.3 ± 0.05 *k*_B_*T*/*e* (−33 ± 1 mV), −1.6 ± 0.11 *k*_B_*T*/*e* (−41 ± 3 mV), and −1.5 ± 0.04 *k*_B_*T*/*e* (−39 ± 1 mV) for SLBs nominally containing 5, 10, and 20% of POPG respectively. (c) Comparison of measured (symbols) and calculated (solid lines) values for ln[ln(*t*_esc_/*t*_r_)] reveals good agreement over the whole range of *κh*. Calculated ln[ln(*t*_esc_/*t*_r_)] values were obtained using eq 4 where ϕ_s_ values were taken from Δ*F*_el_ vs *κh* fits in (b). (d) Comparison of relative POPG fraction in the obtained SLBs with that in the precursor lipid mixtures reveals the absence of a strong correlation. We infer 1–2% charged lipid in the SLBs with nominal POPG fractions of 5–20%. (e) Representative calculation of electrostatic potential in the slit obtained by solving the Poisson–Boltzmann equation for a given device geometry; solution conditions (2*h* = 68.4 nm, pH 5.85 and *c* = 1.10 mM) and surface charge density given by eq 5 with the number density of POPG phosphate groups Γ = 0.021 nm^–2^.

**Figure 5:**
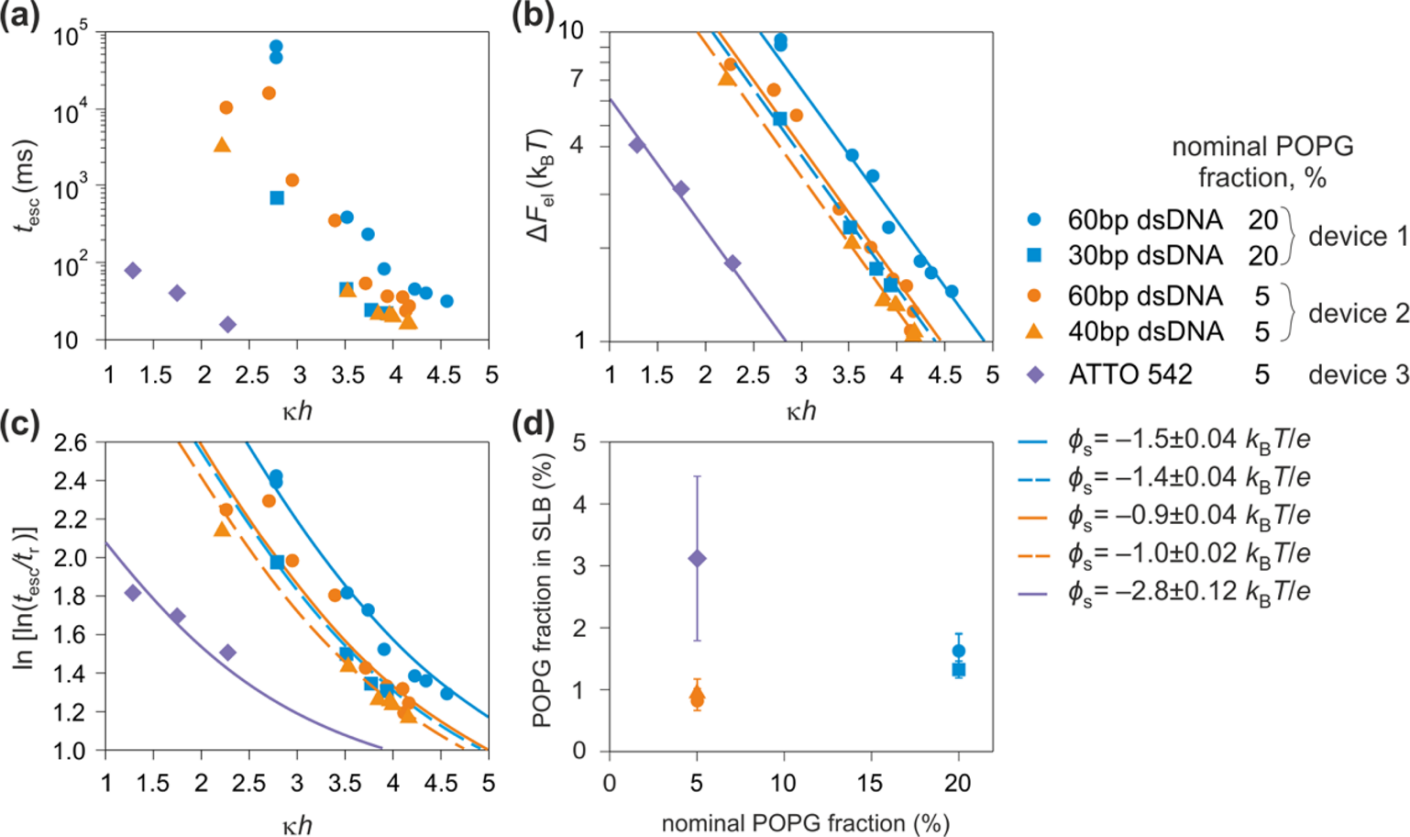
Trapping 60bp dsDNA (*q*_eff_ = −44.8 *e*, circles), 40bp dsDNA (*q*_eff_ = −33.8 *e*, triangles), 30bp dsDNA (*q*_eff_ = −28.1 *e*, squares), ATTO 542 (*q*_eff_ = −3.0 *e*, diamonds) molecules in three different nanoslit SLB devices. Data are provided for measurements on SLBs nominally containing 20% of POPG (blue symbols and lines; device 1), and 5% of POPG in different devices (orange and violet symbols and lines; devices 2 and 3). (a) Dependence of measured escape times, *t*_esc_, on *κh*. (b) Measured values (symbols) for electrostatic free energy Δ*F*_el_ fitted to the eq 3 with a single fit parameter, ϕ_s_. We obtained ϕ_s_ = −1.5 ± 0.04 *k*_B_*T*/*e* (−39 ± 1 mV) and −1.4 ± 0.04 *k*_B_*T*/*e* (−36 ± 1 mV) for 60bp (solid blue line) and 30bp dsDNA (dashed blue line) in the SLB system nominally containing 20% of POPG (device 1). For measurements with nominal 5% of POPG we obtain −0.9 ± 0.04 *k*_B_*T*/*e* (−23 ± 1 mV) and −1 ± 0.02 *k*_B_*T*/*e* (−26 ± 0.05 mV) for 60bp (solid orange line) and 40bp dsDNA (dashed orange line; device 2) and −2.8 ± 0.12 *k*_B_*T*/*e* (−72 ± 3 mV) for ATTO 542 (violet solid line; device 3). (c) Comparison of measured (symbols) and calculated (solid lines) values for ln[ln(*t*_esc_/*t*_r_)] reveals good agreement over the whole range of *κh*. Calculated ln[ln(*t*_esc_/*t*_r_)] values were obtained using eq 4 where ϕ_s_ values were taken from Δ*F*_el_ vs *κh* fits in (b). (d) Comparison of relative POPG fraction in the SLBs with that in the precursor lipid mixtures indicates ≈1–3% negatively charged lipids in the SLBs with nominal POPG fractions of 5 and 20%.

In order to probe the ability to tune the surface charge density using SLB composition, we performed a series of ET*e* measurements using devices passivated with SLBs derived from SUVs of various lipid compositions (Figures 4 and 5). We investigated three different lipid compositions with nominal POPG content (as determined by the mixing ratio of POPG and POPC in the precursor lipid mixtures) of 5, 10, and 20 mol %. In the first series of experiments, we performed measurements of escape-time, *t*_esc_, for the same molecular species, namely 60bp dsDNA end-labeled with two ATTO 532 dye molecules (Figure 4a). We used the same trapping device sequentially passivated with lipid bilayers containing nominally different relative fractions of POPG. Prior to every new passivation procedure, we removed the previous SLB using a 10% solution of the surfactant sodium dodecyl sulfate (SDS). Doing so permitted us to compare SLBs of various composition keeping slit geometry identical. Interestingly, we obtained similar escape time values for all of the probed lipid bilayer compositions (Figure 4a). Since the value of *t*_esc_ for a given molecule at a particular *κh* value is a function of effective surface electrical potential, ϕ_s_, alone, the observation indicates that all probed SLBs had similar surface electrical properties as relevant for long-ranged electrostatic interactions.

In order to quantify the values of ϕ_s_ in each experiment and to infer the corresponding percentage of negative POPG lipids in the SLBs, we first converted the measured escape time values, *t*_esc_, to electrostatic interaction free energies, Δ*F*_el_, as described in our previous work.^35^ We then plotted the inferred values Δ*F*_el_ values vs *κh* and fitted the data to the following equation:
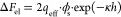
3with a single fit parameter, ϕ_s_. Here we use eq 2 and the relation Δ*F*_el_ = *q*_eff_ ϕ_m_, established in previous experimental and theoretical work.^35,43^ Furthermore, we use the calculated effective electrical charge, *q*_eff_ = −44.8 *e*, for labeled 60bp dsDNA which has been verified in previous measurements.^35,37−40^ κ and *h* are in turn known from conductivity measurements of salt concentration in the measurement solution and from AFM measurements of slit height, respectively. Measurements of escape times of 60bp dsDNA in nanostructured SLBs thus yielded ϕ_s_ values in a range from −1.3 *k*_B_*T*/*e* (−33 mV) to −1.6 *k*_B_*T*/*e* (−41 mV) for nominal POPG fractions from 5 to 20% (Figure 4b). We did not observe strong correlation between the measured surface potentials and the percentage of negative lipid content in the precursor lipid mixtures. Moreover, the inferred values of ϕ_s_ are about a factor of 2 smaller than the corresponding values of ϕ_s_ = −2.7 *k*_B_*T*/*e* (−69 mV) and −3.4 *k*_B_*T*/*e* (−87 mV) calculated using the Poisson–Boltzmann electrostatics model for SLBs nominally containing 5 and 20% of POPG respectively, at pH 5.8 and *c* = 1.2 mM as described later.

Next, in order to further characterize the reproducibility of the coating procedure as well as the stability and longevity of the SLB coatings in our work, we performed similar measurements and analysis, as described above, for few different molecular species in three different lipid bilayer-passivated devices (Figures 5a,b). Specifically, we used: (1) 60bp and 30bp dsDNA, (2) 60bp and 40bp dsDNA, and (3) ATTO 542 dye molecules in devices passivated with lipid bilayers nominally containing either 5 or 20% of POPG (Figure 5). Similar to 60bp dsDNA, both the 30bp and 40bp dsDNA molecular species were labeled with two ATTO 532 dyes, and the *q*_eff_ values for all dsDNA molecules were taken from the measurements and calculations performed as described in earlier work.^40,43^ In particular, we used the values −28.1 *e*, −33.8 *e*, and −44.8 *e* for 30bp, 40bp, and 60bp dsDNA, respectively.^40^ The value of *q*_eff_ for ATTO 542 was assumed to be equal to its structural charge, which is −3 *e*. Fitting the measured Δ*F*_el_ vs *κh* data to eq 3, we obtained similar fit values for ϕ_s_ for both pairs of molecules measured in the same devices, as expected (Figure 5b, devices 1 and 2). Specifically, *t*_esc_ measurements on 60bp and 30bp dsDNA molecules in SLB structures with nominally 20% of POPG yielded values of ϕ_s_ = −1.5 ± 0.04 *k*_B_*T*/*e* (−39 ± 1 mV) and −1.4 ± 0.04 *k*_B_*T*/*e* (−36 ± 1 mV), and *t*_esc_ measurements on 60bp and 40bp dsDNA molecules in SLB structures with nominally 5% of POPG yielded ϕ_s_ = −0.9 ± 0.04 *k*_B_*T*/*e* (−23 ± 1 mV) and −1.0 ± 0.02 *k*_B_*T*/*e* (−26 ± 0.5 mV), respectively (Figure 5b, devices 1 and 2). Good agreement of ϕ_s_ values obtained for different molecules in the same system shows that a precise measurement of *t*_esc_ for one calibrator molecule, i.e., a molecule with the known value for *q*_eff_, is sufficient to accurately determine ϕ_s_, which may then be applied to the determination of *q*_eff_ for a test molecular species of unknown effective charge. Interestingly, our experiments on 60bp dsDNA, measured in two different SLB nanoslit devices, composed of SLBs containing nominally identical POPG fraction of 5%, revealed escape times with disparities of around 50% under the same measurement conditions, characterized by the value of the system size parameter *κh* (Figure S1). These *t*_esc_ values measured in two different devices correspond to inferred surface electrical potential values of ϕ_s_ = −1.3 ± 0.05 *k*_B_*T*/*e* (−33 ± 1 mV) and −0.9 ± 0.04 *k*_B_*T*/*e* (−23 ± 1 mV) for two nominally identical SLBs (Supporting Section 1, Figures 4b and 5b, device 2). Approximately 15–25% of the observed disparity in *t*_esc_ values, and consequently ≈6–12% in ϕ_s_ values, could however stem from small inaccuracies in the estimate of the relevant *κh* value (Supporting Section 1). We attribute the residual discrepancy in escape times for the same molecular species to ≈30% difference in the surface charge density of the SLBs in the two experiments. Importantly, using ATTO 542 as a probe molecule, we did note a higher magnitude of surface potential, ϕ_s_ = −2.8 *k*_B_*T*/*e* (−72 mV) for measurements under similar experimental conditions in a third device coated with 5% POPG. This value suggests a percentage of POPG in the bilayer closer to the nominal value of 5% (Figure 5d). Assuming the nature of the probe molecule has no impact on the measured interaction, our findings suggest that SLBs prepared from precursor lipid mixtures of identical compositions can differ in content of negative POPG lipids and therefore display variable surface electrical potentials. Our observations emphasize the necessity for a calibration measurement to determine ϕ_s_ for an SLB system, with the composition variabilities from one SLB device to the next likely arising from interactions between the solid silicon dioxide support and lipid vesicles which is not highly controllable.

We then investigated the temporal stability of SLB surface properties by performing repeated measurements of *t*_esc_ on the same molecular species at similar *κh* values over more than 24 h (Supporting Section 2). The absence of significant variation in measured escape times indicates that obtained bilayers are stable in composition for at least 24 h. Thus, despite possible compositional variability of SLBs from one device to the next, we establish that the electrostatic properties of SLBs, probed using electrostatic trap performance in a nanostructured SLB system, display high temporal stability in a given measurement device.

Further, in order to compare the dependence of *t*_esc_ on *κh* measured experimentally, with that predicted by Kramer’s relation given by eq 1, we plotted the experimentally measured and theoretically predicted values for ln[ln(*t*_esc_/*t*_r_)] vs *κh* (Figures 4c and 5c). Combining eq 1 with the relation for *W* = Δ*F*_el_ + *f* = *q*_eff_ ϕ_m_ + *f*, we obtained the following expression for theoretically predicted ln[ln(*t*_esc_/*t*_r_)] values:
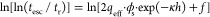
4

The trend observed for ln[ln(*t*_esc_/*t*_r_)] vs *κh* is not linear due to the presence of the *f* term on the right-hand side (RHS) of the equation. In order to calculate the RHS of eq 4, we used the ϕ_s_ values obtained from Δ*F*_el_ vs *κh* fits (Figures 4b and 5b) and *q*_eff_ values for molecules taken from calculations.^43^ Note that *f* is a fluctuation free energy that depends on device geometry and can be calculated for a given set of experimental conditions as previously described.^38^ To estimate the experimental ln[ln(*t*_esc_/*t*_r_)] values, we used the measured *t*_esc_ values (Figures 4a and 5a) and an estimate for the position relaxation time of the molecule given by *t*_r_ ≈ *L*^2^/4*D*. Here *L*≈ 450 nm is an effective diffusion length within the trap, estimated from BD trajectory simulations, *D* = *k*_*B*_*T*/6*πηr*_H_ is the diffusion coefficient, where *η* denotes water viscosity, and *r*_H_ is the hydrodynamic radius of the object. For instance, for 60bp dsDNA, we obtained *t*_r_ ≈ 1 ms using *r*_H_ = 4.51 nm measured using fluorescence correlation spectroscopy (FCS), as described in our previous work.^35,40^ We observe good agreement between calculated and measured ln[ln(*t*_esc_/*t*_r_)] values over the whole range of tested *κh* values.

### Inferring the Charge Content of SLBs in Nanoslits from ET*e* Measurements

Finally, we outline the theoretical model used to relate values of effective surface electrical potential, ϕ_s_, inferred from experiment to a percentage of negatively charged POPG lipids in the SLBs (Figures 4d and 5d). We modeled the lipid bilayer surfaces in our system using a single p*K*_a_ charge regulation model to describe the electrical charge density of the surface as a function of salt concentration, pH and number density of ionizable groups, Γ.^35,37,52^
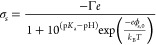
5where *K*_a_ is acid dissociation constant of the phosphate groups in POPG, and we assume p*K*_a_ = 3.5 (refs (52−54)). Equation 5 serves as a boundary condition for the Poisson–Boltzmann (PB) equation, Δ*ψ* = *κ*^2^ sinh*ψ*, describing the spatial distribution of the dimensionless electrostatic potential, , in the system. The PB equation was solved for a range of input values for Γ in eq 5 yielding corresponding values of the true surface potential, ϕ_s,0_ (Figure 4e). As mentioned previously ϕ_s,0_ is generally larger than the effective surface potential, *ϕ*_s*,*_ accessible in our experiments. For example, values of ϕ_s_ = −2.7 *k*_B_*T*/*e* (−69 mV) and −3.4 *k*_B_*T*/*e* (−87 mV) expected for 5% and 20% POPG correspond to ϕ_s,0_ values of −3.2 *k*_B_*T*/*e* (−82 mV) and −5 *k*_B_*T*/*e* (−129 mV), respectively. Our inferred values of ϕ_s_ enable us to infer a surface density of POPG phosphate groups, Γ, in the SLBs in the experiments. Using the literature value for POPG lipid area, *S*_POPG_= 0.7 nm^2^, we converted the obtained Γ values to percentage of POPG lipids in the SLB, *w* (ref (47)). Figures 4d and 5d show the dependence of the inferred value of *w* on percentage of POPG in the corresponding precursor lipid mixture. In general, we observe a range of about 1–3% charged lipids in the SLBs with nominal POPG fractions of 5–20% in all our experiments. Moreover, measurements with two different molecules in the same SLB-coated device confirm that *w* is relatively independent of the type of probe molecule. Specifically, measurements on 40bp and 60bp dsDNA in the same SLB structure with a nominal concentration of 5% POPG yielded values of *w* = 1.0 ± 0.2% and 0.8 ± 0.2%, and measurements on 30bp and 60bp dsDNA in 20% POPG SLBs yielded *w* = 1.3 ± 0.1% and 1.6 ± 0.3%, respectively (Figures 5d, devices 1 and 2).

Thus, contrary to intuitive expectations, we did not observe a strong correlation of POPG content in the probed SLBs with that in the lipid mixtures used for vesicle preparation. For all compositions studied, the nanoslit SLBs displayed a lower fraction of POPG compared to nominal POPG content (as determined by the mixing ratio of POPC and POPG in the precursor lipid mixture). The significant disparity in lipid compositions of SLBs compared to the precursor lipid mixtures has in fact been repeatedly reported in previous work.^55−62^ Possible origins for this effect may lie in specific interactions between the lipid bilayer and solid support, surface-induced impeded lipid mobility, intermembrane repulsion between neighboring charged lipid head groups, as well as heterogeneity in lipid vesicle suspensions.^57,62^ Similar observations have been previously reported (ref (62)) by Gilbile et al., who measured surface electrical potentials for SLBs using the surface force apparatus (SFA) in a solution of low salt concentration (0.5 mM NaNO_3_, pH 5.7). Surface potential measurements in that study suggested the presence of ≈1% of anionic charged dimyristoylphosphatidylglycerol (DMPG) lipids in lipid bilayers prepared using precursor lipid mixtures containing 10 and 20% of DMPG.^62^ These experiments using macroscopic surfaces strongly echo our own observations involving molecular scale probes of the electrostatic interaction in solution.

One possible explanation for the observed paucity of POPG in the SLBs in our experiments could be related to the fact that lipid vesicles, prepared by extrusion through 30 nm pore filters, are likely highly heterogeneous in size and lipid composition. Previous characterization of extruded vesicles by electron microscopy and dynamic light scattering have shown that extrusion of multilamellar lipid vesicles (MLV) through 30 nm pores results in an average vesicle size of ≈60 nm in diameter with standard deviation of ≈20 nm (refs (63, 64)). It is also known that small vesicles (≈ 50 nm in diameter) can have a high degree of heterogeneity, i.e., up to 10-fold variation in relative lipid composition, at the single vesicle level.^65^ It is thus possible that the SLBs in our nanoslits form from a fraction of SUVs that contain comparatively low amounts of negatively charged POPG lipids. Moreover it is likely that electrostatic repulsion between negative SUVs and silicon dioxide surfaces—though small at pH 3.5 and salt concentration 100 mM—may play a further role in “electrostatic sieving” of the SLB forming vesicles, presenting a slightly lower energy barrier to entry into the nanoslits for vesicles with a smaller relative fraction of POPG. Furthermore, in a nanoscale system where the Debye length, κ^–1^, is comparable to a critical system dimension (in this case, the gap between two facing, charged bilayers, *h*), the system could minimize its electrical free energy by a gradual change in composition occurring by diffusive draining of charged lipids out of the narrow parallel-plate slit region. The observed depletion of negatively charged POPG in the obtained SLBs could thus arise from (1) lower POPG content in the vesicles entering the nanoslits and/or forming the nanoslit SLB and (2) redistribution/drainage of charged lipids in the SLB out of the nanoslit region.

## Conclusions

In conclusion, we have characterized the electrical properties of charged lipid bilayers in a nanofluidic system. We have demonstrated the use of lipid bilayer surface coatings formed by vesicle fusion for stable electrostatic trapping of charged molecules in solution. The highly acidic phosphate group of POPG supports strong electrostatic interactions in nonbuffered aqueous solutions (pH 5–6). Although we have focused on negatively charged molecules and surfaces in this work, the same principles may be applied to generate positively charged lipid bilayers in order to trap positively charged molecules in solution. Whereas the charge composition of SLBs was found to vary significantly between devices, we found that SLBs displayed good temporal stability in surface electrical properties over a period of 24 h within a given device. The results of this study carry broad implications for the use of charged lipid bilayers as surface coatings in a variety of experiments and measurement situations in nanoscience and nanotechnology.

## Supporting Information Available

Additional experimental details and discussion of the temporal stability of electrostatic trapping and device longevity in a nanostructured SLB system, device-to-device variation of lipid bilayer-coated devices, list of symbols and abbreviations (PDF)
Supporting movie displaying SLBs circumventing the pocket structures (AVI)

## Supplementary Material

la2c02203_si_001.pdf

la2c02203_si_002.avi
