# Supplementary material for: Single-Molecule Trapping and Measurement in a Nanostructured Lipid Bilayer System
Source: Langmuir. 2022 Nov 3;38(45):13923–34. doi: 10.1021/acs.langmuir.2c02203 (PMC9671048; doi:10.1021/acs.langmuir.2c02203)
Supplement: Supplementary file 1 — la2c02203_si_001.pdf [file la2c02203_si_001.pdf]

Single-Molecule Trapping and Measurement in a Nanostructured Lipid Bilayer System

Supporting Information

Maria Bespalova[1]*, Robin Öz*[2]*, Fredrik Westerlund*[2] *& Madhavi Krishnan*[1,3]*

[1]Physical and Theoretical Chemistry Laboratory, Department of Chemistry, University of Oxford, South Parks Road, Oxford OX1 3QZ, United Kingdom

[2]Department of Biology and Biological Engineering, Chalmers University of Technology, Gothenburg, Sweden

[3]The Kavli Institute for Nanoscience Discovery, Sherrington Road, Oxford OX1 3QU, United Kingdom

Section 1. Exploring the dependence of device-to-device variation on SLB composition

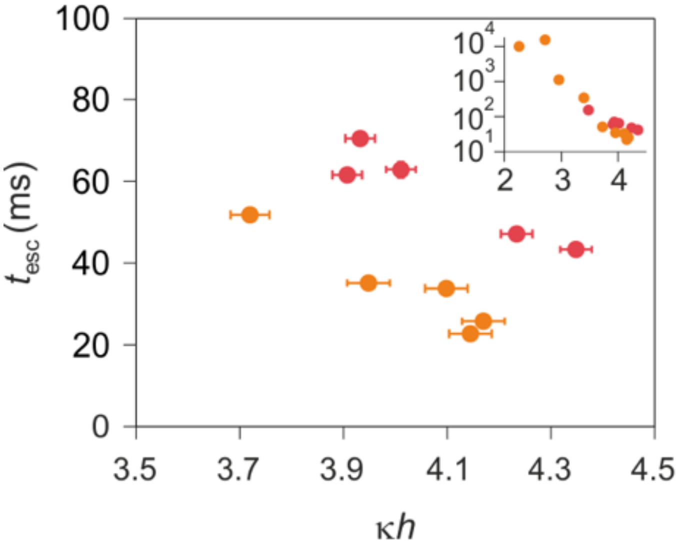

Figure S1. Escape time measurements on 60bp dsDNA molecular species in SLB nanostructures formed in two different devices (depicted with red and orange colors). Both SLB nanostructures contain nominally identical relative POPG fraction of 5%. The errors on t_{esc} ($< 2.5\%$) are smaller than the data symbols.

In order to explore the dependence of device-to-device variation on SLB composition we performed t_{esc} measurements for the same molecular species, i.e., 60bp dsDNA, in two different devices coated with bilayers under nominally identical conditions. The SLBs were prepared from precursor lipid mixture containing identical relative POPG fraction of 5%. Although we followed the same passivation and measurement procedures, we found that the obtained t_{esc} values varied within up to ≈50% under the same measurement conditions, characterized by the value of the system size parameter κh (Figure S1). Here κ^{-1} denotes the Debye length and $h = h_{\mathrm{s}} - 4$ (Figure 1b)[1]. However it is also possible that part of the observed discrepancy could stem from slightly inaccurate κh estimation for one or both cases. As we have shown in our previous work, one of the main contributions to the measurement error arises from the uncertainty on the slit height, $2h_{\mathrm{s}}$, measured by AFM[2,3]. For instance, in the case depicted in Figure S1 over- or underestimation of $2h_{\mathrm{s}}$ by 1-2 nm for the dataset shown with red or orange color respectively would explain 15-25% of observed t_{esc} discrepancy. Taking this into consideration it is unlikely that the observed difference in performance between the two devices arises solely from the properties of the SLBs.

Section 2. Probing the temporal stability of electrostatic trapping and device longevity in a nanostructured SLB system

In order to probe the stability of SLB coatings, and consequently their applicability for reproducible and stable electrostatic trapping, we performed repeated t_{esc} measurements for 40bp and 60bp dsDNA molecular species within 24-48 hours in the same SLB nanostructure. Figure S2 depicts up to ≈5% discrepancy in measured t_{esc} values for 40bp dsDNA and ≈23% discrepancy for 60bp dsDNA. In the latter case the larger discrepancy can be explained by slightly different κh values that cannot always be tightly controlled due to slight salt concentration drifts that are possible during a given measurement. From the perspective of ϕ_{s} measurements however, a 23% departure in measured escape time would correspond to a ≈6%

error on measured ϕ_s value for 60bp dsDNA ($q_{\rm eff} = -44.8\ e$), since the fractional error on ϕ_s when $q_{\rm eff}$ is known (or equivalently on $q_{\rm eff}$ when ϕ_s is known) is expected to be $\approx t_{\rm esc,e} k_B T/\Delta F_{\rm el}$ (Ref. 2, 4, 5). Here $t_{\rm esc,e}$ is the fractional measurement error on $t_{\rm esc}$ and $\Delta F_{\rm el} = q_{\rm eff}\phi_m = 3.8\ k_B T$ in this case. For the measurements on 40bp dsDNA ($q_{\rm eff} = -33.8\ e$), a 5% departure in measured $t_{\rm esc}$ and an inferred value of $\Delta F_{\rm el} = 3.5\ k_B T$ would correspond to a ≈1.4% error on measured ϕ_s, which is relatively small.

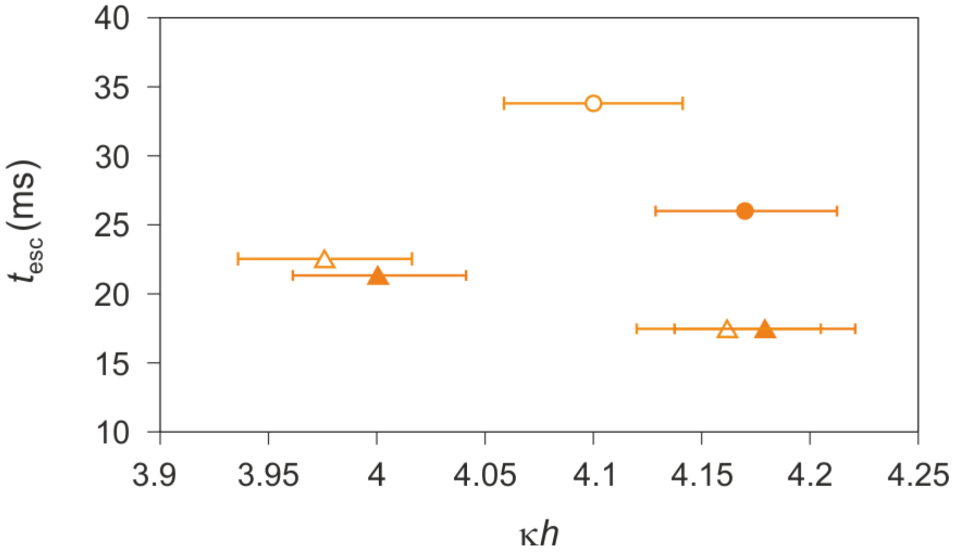

Figure S2. Repeated $t_{\rm esc}$ measurements using 40bp dsDNA (triangles) and 60bp dsDNA (circles) molecular species in the same SLB nanostructure device. Open symbols represent the values for $t_{\rm esc}$ obtained in 24-48 hours after the initial measurements depicted with filled symbols. The errors on $t_{\rm esc}$ ($< 1\%$) are smaller than the data symbols.

Supporting Movie

The video displays SLBs traversing neighboring ≈5 μm wide slits and circumventing the nanostructured circular indentations of radii, r≈250 and ≈370 nm, while flushing a solution of SUVs at pH 3.7 and containing c =100 mM NaCl, prepared from 20% of POPG. The sampling rate in the experiment was 0.4 Hz. The video is sped up by a factor 25.

List of symbols and abbreviations

AFM	Atomic force microscopy
bp	Basepair
c	Salt concentration
d	Depth of the pocket structure
ds	Double stranded
D	Diffusion coefficient
δ	Thickness of a single lipid bilayer
ETe	Escape-time electrometry
e	Elementary unit charge
f	Fluctuation free energy
ΔF_{el}	Electrostatic interaction free energy
Γ	Number density of ionizable groups
$2h_{\mathrm{s}}$	Slit height measured by AFM
$2h$	Effective height of a slit (=$2h_{\mathrm{s}} - 2\delta$)
I_0	Intensity at the feature-free flat surface region of a slit
I_{p}	Intensity at the pocket locations
K_{A}	Acid dissociation constant
κ^{-1}	Debye length
k_{B}	Boltzmann's constant
MLV	Multilamellar vesicles
N	Number of recorded escape events
N_{A}	Avogadro's number
η	Solvent viscosity
POPC	1-palmitoyl-2-oleoyl-glycero-3-phosphocholine
POPG	1-palmitoyl-2-oleoyl-sn-glycero-3-phospho-(1'-rac-glycerol) (sodium salt)
q_{eff}	Effective electrical charge
r_{H}	Hydrodynamic radius
SLB	Supported lipid bilayer
SUV	Small unilamellar vesicles
σ_s	Electrical charge density of the surface
T	Temperature
Δt	Duration of an individual molecular escape event
t_{esc}	Average escape time of the molecule
t_{r}	Position relaxation time of the trapped molecule
W	Depth of the trap or potential well
x_{e}	Error on parameter x
ϕ_{m}	Electrical potential at the slit mid-plane
ϕ_{s}	Effective surface electrical potential
$\phi_{\mathrm{s},0}$	True surface electrical potential

References

1. Rog, T.; Murzyn, K.; Pasenkiewicz-Gierula, M., Molecular dynamics simulations of charged and neutral lipid bilayers: treatment of electrostatic interactions. *Acta Biochimica Polonica* **2003,** *50* (3), 789-798.
2. Ruggeri, F.; Zosel, F.; Mutter, N.; Rozycka, M.; Wojtas, M.; Ozyhar, A.; Schuler, B.; Krishnan, M., Single-molecule electrometry. *Nature Nanotechnology* **2017,** *12* (5), 488-495.
3. Ruggeri, F.; Krishnan, M., Spectrally resolved single-molecule electrometry. *Journal of Chemical Physics* **2018,** *148* (12), 123307.
4. Ruggeri, F.; Krishnan, M., Lattice diffusion of a single molecule in solution. *Physical Review E* **2017,** *96* (6), 062406.
5. Ruggeri, F.; Krishnan, M., Entropic Trapping of a Singly Charged Molecule in Solution. *Nano Letters* **2018,** *18* (6), 3773-3779.
